# From Numerical Taxonomy to Classifier Modeling: A Quantitative Taxonomic Workflow for *Euterpnosia* Cicadas (Hemiptera: Cicadidae)

**DOI:** 10.3390/insects17090899

**Published:** 2026-08-27

**Authors:** Tung-Yu Hsieh, Feng Li

**Affiliations:** 1 School of Food & Bioengineering, Fujian Polytechnic Normal University, Fuqing 350300, China; 2 Fujian Universities and Colleges Engineering Research Center of Modern Facility Agriculture, Fujian Polytechnic Normal University, Fuqing 350300, China; 18850659211@139.com; 3 Wuyou Ecological Agriculture Co., Ltd., Fuqing 350300, China; 4 Fujian Tianyuan Shiguang Leisure Agriculture Co., Ltd., Fuqing 350300, China; 5 Lanxi Agricultural Technology Co., Ltd., Fuqing 350300, China; 6 Cai Die Biological Technology Co., Ltd., Yongtai 350700, China; 7 Key Laboratory of Genetics, Breeding and Multiple Utilization of Crops, Ministry of Education, Fujian Agriculture and Forestry University, Fuzhou 350002, China; 8 Key Laboratory of Biological Breeding for Fujian and Taiwan Crops, Ministry of Agriculture and Rural Affairs, Fujian Agriculture and Forestry University, Fuzhou 350002, China

**Keywords:** Cicadidae, classifier modeling, numerical taxonomy, phenetics, character selection, quantitative key, reject option, reference library, type material

## Abstract

Numerical taxonomy reveals morphological structure, whereas classifier modeling tests identification against labeled reference hypotheses. We evaluated 70 Taiwanese *Euterpnosia* Matsumura, 1917 specimens representing five operational classes, using 71 external characters for unsupervised analysis and 20 non-destructive characters for supervised modeling. Broad character retention was separated from goal-specific selection: taxonomists may prespecify candidates from literature or experience, while data-driven selection remained inside training folds. In 20 × five-fold nested cross-validation, feature-screened multinomial accuracy was 95.93% (balanced accuracy 94.71%); the all-character model reached 96.57%, showing that selection need not force parsimony when a compact pool is already informative. Leave-one-species-out tests rejected omitted *E. chilanensis* Chen, Hsieh, Chen, Chen & Chang, 2021, *E. olivacea* Kato, 1927, and *E. hoppo* Matsumura, 1917 in 100%, 100%, and 92.0% of decisions, but *E. alpina* Chen, 2005 and *E. varicolor* Kato, 1926 only 21.1% and 35.0%. CART selected X49, X20, and X32 for a concise quantitative-key draft. The workflow can prioritize candidate diagnostic characters and support identification, abstention, and key construction, but does not independently establish species boundaries or nomenclatural conclusions.

## Simple Summary

Taxonomists spend substantial effort finding morphological characters that reliably distinguish species. We evaluated a workflow that links numerical taxonomy with classifier modeling, meaning the construction and validation of statistical classifiers from named reference specimens. Broad sets of external characters are first used to explore morphological structure. For practical identification, taxonomists can then define a smaller candidate set from literature and experience, and algorithms can test which characters or combinations are most useful. Across 70 Taiwanese *Euterpnosia* cicadas, specimens from five operational reference classes were classified with high out-of-sample accuracy. A 20-character classifier reached 96.57% accuracy, whereas a classification tree selected only three characters to produce a simpler draft key with lower accuracy. When one species was deliberately omitted from the reference library, reliable rejection occurred only when its morphology was sufficiently separated from represented classes. The workflow can therefore help taxonomists prioritize diagnostic characters, build identification models and quantitative keys, and recognize uncertain assignments. It does not replace type study, species delimitation, nomenclature, or expert taxonomic judgment.

## 1. Introduction

Taxonomy supplies the names, diagnoses, and organismal hypotheses on which biodiversity science depends, yet reliable identification increasingly exceeds available specialist capacity. This taxonomic impediment reflects declining expertise, limited support for revisions, incomplete access to collections, and the time required to compare variation across specimen series [1,2,3,4,5]. Misidentifications can propagate through collections and downstream biodiversity analyses [6,7]. Quantitative methods are therefore most useful when they formalize repeatable components of expert practice while preserving the distinction among morphological pattern discovery, specimen identification, species delimitation, phylogenetic inference, and nomenclature.

Modern taxonomy comprises distinct but connected tasks—discovery, delimitation, diagnosis, description, and specimen determination—and no classifier substitutes for all five [8]. Numerical taxonomy, or phenetics, describes overall resemblance in a defined character space. Phylogenetic systematics addresses historical relationships and character transformation. These are different inferential tasks: a stable phenetic group is neither a clade nor, by itself, a species. Nevertheless, phenetic structure can provide a reproducible descriptive hypothesis, guide the search for diagnostic characters, and identify specimens that require further taxonomic study. Species hypotheses arise through biological and causal reasoning and remain open to tests using morphology, types, calling songs, geography, ecology, and molecular evidence where available [9,10]. We therefore use unsupervised morphology for pattern discovery and supervised models for diagnosis and specimen determination, not as a substitute for phylogeny or total-evidence delimitation.

Quantitative external morphology offers a complementary route that remains closely connected to traditional characters. Homologous dimensions can be measured directly from preserved specimens or living individuals, or extracted from images after the relevant anatomical landmarks have been defined consistently. Instead of treating every image cell as an independent signal, landmark-derived distances and ratios compress anatomically interpretable information into a smaller feature set. Dimensionless ratios between homologous distances are unchanged by uniform image scaling, so a physical scale bar is not required when relative shape alone is used; calibrated images additionally permit absolute linear dimensions to be recovered. This reduces dimensionality, permits taxonomists to add characters emphasized in descriptions or prior revisions, and retains an explicit link between algorithmic output and observable morphology. Morphometric and machine-learning studies have distinguished insects using outlines, landmarks, or routine measurements [11,12,13,14,15,16], while image-centered systems now operate across large and heterogeneous arthropod libraries [17,18,19,20]. Accordingly, the workflow remains centered on observable taxonomic morphology.

Character selection is itself a practical taxonomic task. During unsupervised phenetic discovery, a broad homologous character set is desirable because premature filtering can hide morphological structure that was not anticipated a priori. Once operational reference groups are established, however, the objective changes from describing overall resemblance to building an efficient diagnostic rule. At that stage, a compact set or combination of accessible, reproducible, and discriminating characters can reduce the measurement burden, simplify models and keys, and, in some data sets, improve generalization by excluding redundant or noisy variables. Taxonomists have traditionally performed this selection from comparative experience and the literature; earlier work on classifier modeling formalized the same idea through model-variable selection [21], while morphometric work on *Euterpnosia* ranked numeric diagnostic characters by their separation and reliability [22]. The present workflow therefore treats expert knowledge and algorithmic selection as complementary: a taxonomist may define an a priori candidate pool, after which model-based selection is performed only within training data. Selected variables are candidate diagnostic characters for expert interpretation, not automatic evidence of species status, phylogenetic significance, or synapomorphy.

The workflow developed here follows a taxonomy-oriented sequence. First, a broad character set is analyzed without class labels to determine whether reproducible morphological groups occur and whether their structure remains stable under specimen and character perturbation. Second, those operational groups are interpreted against named reference material and other taxonomic evidence. Third, a practical candidate pool for supervised identification is defined from measurable characters; in other applications, this pool may be prespecified by taxonomists from previous diagnoses, revisionary literature, or experience. Within the training data, multiclass and one-versus-rest models then screen candidate characters, select the number and composition of retained characters, and tune model parameters without access to held-out observations. Fourth, abstention and leave-one-species-out tests identify unsupported assignments and expose incomplete-library failure. Fifth, taxonomically important and name-bearing material is projected for similarity assessment. Finally, a classification tree performs branch-specific character and threshold selection to provide an automatically generated, human-readable first draft of a quantitative key. As an optional extension, a within-group medoid can quantify morphological representativeness when a taxonomist is considering a type candidate for a future new species; it is not a required step in the identification workflow. Formal naming and type designation remain nomenclatural acts performed by taxonomists.

The cicada genus *Euterpnosia* Matsumura, 1917 provides a demanding test. Taiwanese species can differ in calling song while remaining similar in preserved external appearance, and destructive examination is undesirable for types and valuable historical material [23,24,25,26]. Male-centered sampling is common because calling males are more readily detected and collected, and male genitalia and songs provide important evidence. External homologous characters are nevertheless measurable in both sexes, living individuals, and intact specimens. Earlier quantitative work in this research program contributed to the hypothesis later formalized in the conventional description of *E. chilanensis* Chen, Hsieh, Chen, Chen & Chang, 2021 [22]. That history motivates the present workflow but is not treated as independent prospective validation.

Terminology varies among taxonomy, morphometrics, and machine learning. We retain classifier modeling, used across our earlier peer-reviewed studies [21,27], as an umbrella term for constructing, selecting, validating, and applying supervised classifiers to taxonomically defined reference classes. Classifier-assisted identification refers here only to the downstream use of a fitted classifier for specimen determination; it is not a replacement term for classifier modeling. Likewise, character selection refers to choosing taxonomic predictors in biological terms, whereas feature selection denotes the statistical selection step inside model training. These processes can overlap operationally, but a statistically selected feature is not automatically a taxonomically significant character. Earlier applications of classifier modeling emphasized fit to reference data. Contemporary predictive standards require separation of fitting, model selection, and evaluation: feature selection before cross-validation produces optimistic error estimates [28], small data sets require leakage-resistant resampling [29], and model scores require calibration assessment using proper scoring rules and reliability diagnostics [30,31]. Expert knowledge can legitimately define the candidate-character pool before evaluation, but any selection informed by the present labeled data is part of model selection and must remain inside resampling. For uncertain specimens, we use abstention or reject option for withholding assignment when model support is insufficient, and open-set testing for evaluating specimens or taxa absent from the represented reference classes [32,33,34,35,36].

We tested four linked hypotheses. First, the 71-character matrix contains a stable five-group morphological structure under specimen and character perturbation. Second, 20 externally accessible characters support accurate classification of unseen measurements from the same reference domain. Third, abstention trades coverage for reliability, whereas an omitted species is rejected only when sufficiently separated from represented classes in measured morphospace. Fourth, a classification tree can translate multivariate structure into a rapid, auditable first draft of a quantitative identification key. We additionally distinguish generally reusable validation principles—nested validation, specimen-level resampling, abstention, omission testing, and training-only character selection—from taxon-specific landmarks, character definitions, thresholds, reference libraries, and biological interpretation. Medoid-based morphological representativeness is presented separately as an optional aid for future type-candidate consideration rather than as a validated core component of the workflow.

## 2. Materials and Methods

### 2.1. Specimens and Analytical Roles

Specimens and image records originated from field surveys and the National Museum of Natural Science, Taichung, Taiwan, China (NMNS); Taiwan Forestry Research Institute, Taipei, Taiwan, China (TFRI); Department of Entomology, National Taiwan University, Taipei, Taiwan, China (NTU); and the Laboratory of Systematic Entomology, Hokkaido University, Sapporo, Japan (SEHU). Living individuals were captured briefly, observed, positioned without injury, photographed for documentary morphometric measurement, and immediately released at the capture locality. They were not killed, dissected, chemically treated, or subjected to an experimental intervention, and no injury or observable adverse effect resulted from the brief handling. Field localities were outside national parks, nature reserves, protected areas, and other zones subject to collection restrictions. Pinned and type material was photographed without destructive preparation whenever possible. The holotype and all paratypes of *E. chilanensis* are deposited in NMNS; the original description did not publish individual accession numbers [22].

Data roles were assigned before model evaluation. All specimens included in the morphological analyses were fully developed adults that had completed the final molt; developmental-stage differences were therefore not a source of morphological variation in the analyzed dataset. Seventy specimens with complete measurements for 71 characters formed the model-development set. These specimens represented five operational reference classes: *E. chilanensis* Chen, Hsieh, Chen, Chen & Chang, 2021 (class 1), *E. alpina* Chen, 2005 (class 2), *E. varicolor* Kato, 1926 including the holotype of *E. elongata* Lee, 2003 (class 3), *E. olivacea* Kato, 1927 including specimens historically identified as *E. kotoshoensis* Kato, 1925 (class 4), and *E. hoppo* Matsumura, 1917 (class 5). The *E. elongata* holotype and historically identified *E. kotoshoensis* material contributed to reference-class definition and are not independent validation. Class labels were taxonomic hypotheses supplied to prediction, not conclusions produced by it. The series was predominantly male because calling males are more readily detected and collected and because traditional cicada diagnoses emphasize male songs and genitalia. The external homologous characters are technically measurable in females, as illustrated for *E. chilanensis* [22]; however, the reconciled file lacks a complete specimen-level sex field, so female identification performance cannot be estimated. Other collection and acquisition metadata were not classifier inputs; the phenetic and predictive analyses were defined on the morphological taxonomic-character matrix.

The source workbook contained 132 specimen columns. Six records with explicitly uncertain varicolor labels and one record labeled “un-1” were excluded before analysis. After removal of records duplicated in the model-development set, 55 specimens remained in the taxonomic-projection set. These records were selected for their relevance to nomenclature, type-locality interpretation, species revision, or assessment of taxa absent from model development. Projection specimens were never used to rank characters, tune regularization, estimate accuracy, or calibrate rejection.

### 2.2. Morphological Character Acquisition

Dorsal images were obtained using a Canon 6D camera (Canon Inc., Tokyo, Japan) with a Canon EF 100 mm f/2.8L Macro IS USM lens (Canon Inc., Tokyo, Japan), mounted on a copy stand, and landmark coordinates were digitized in tpsDig2 (F. James Rohlf, Stony Brook University, Stony Brook, NY, USA). For absolute linear characters, image calibration permits distances to be expressed in millimeters. For ratio characters, the common image scale cancels mathematically, so the same dimensionless values can be calculated without a physical scale bar provided the homologous landmarks are visible and measured consistently. The broader source material also included external morphological information obtained from preserved specimens and living individuals rather than exclusively from photographs. Thus, the classifier modeling framework operates on defined morphological variables rather than raw pixels or collection metadata, and can combine scale-free ratios with calibrated absolute measurements when available.

**Figure 1:**
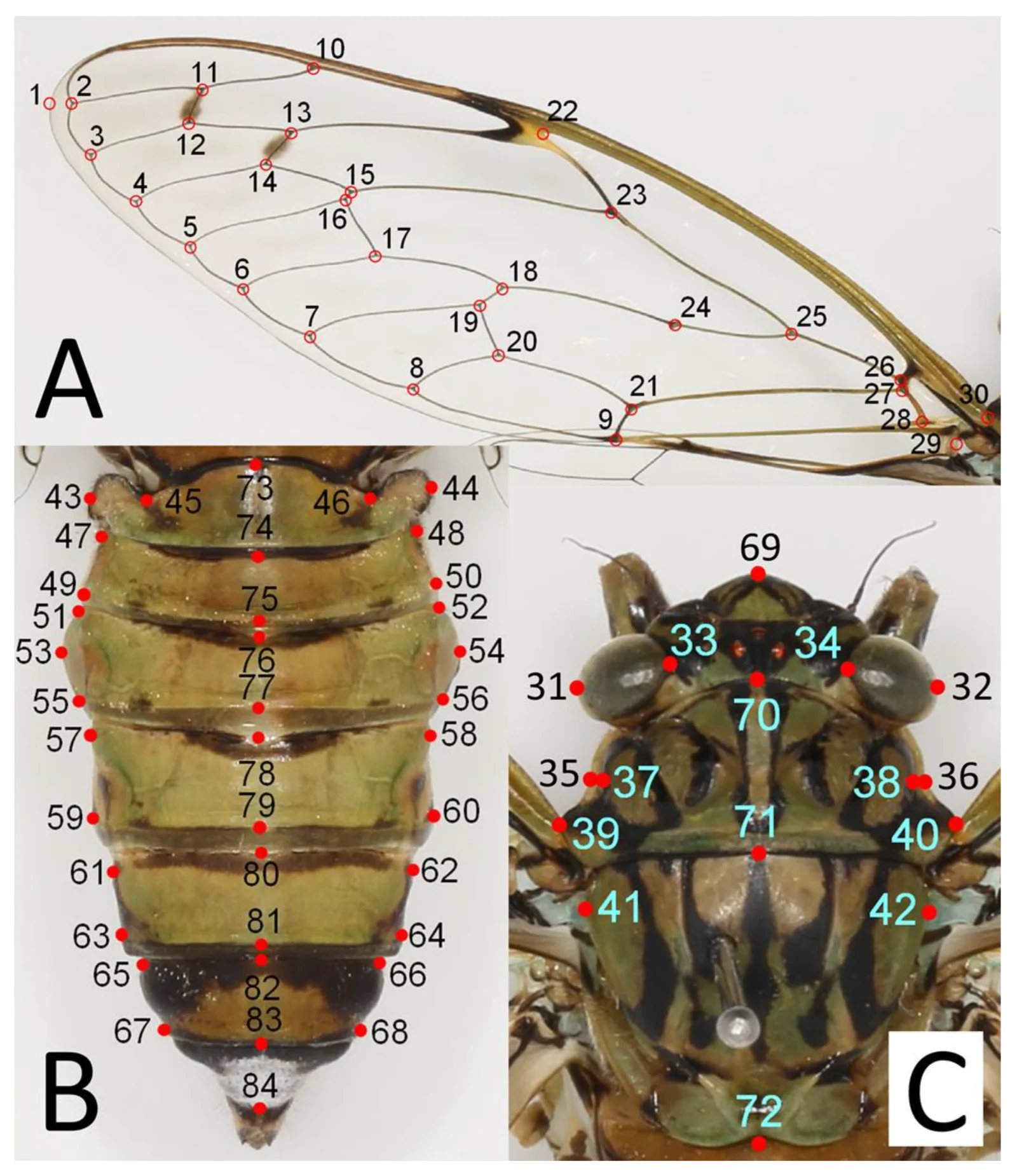
Anatomical landmarks used for *Euterpnosia* morphometrics. (**A**) Forewing landmarks 1–30, shown as open red circles; these require a spread-wing image. (**B**) Abdominal landmarks 43–68 and 73–84, shown as solid red circles. (**C**) Head-and-thorax landmarks 31–42 and 69–72; cyan numerals distinguish the central head-and-thorax series from the red peripheral points. Characters X20–X35, X49, X50, X70 and X71 can be measured without spreading the wings; complete definitions are provided in Table S1. Adapted with permission from Chen et al. (2021), Formosan Entomologist, 41: 192–207 [22].

Eighty-four homologous anatomical landmarks defined the character system (Figure 1). Seventy-one distances or ratios represented the forewing, head and thorax, abdomen, or ratios spanning anatomical regions; absolute distances are expressed in millimeters and ratios are dimensionless. Among the 20 non-destructive predictive characters, 17 are dimensionless ratios, and three (X20, X33, and X49) are absolute distances. Because a common multiplicative image scale cancels in a ratio, the 17 ratio characters describe relative morphology without requiring knowledge of absolute specimen size. The three absolute distances were retained as additional potentially diagnostic information where calibrated measurements were available. No sex correction was applied. Because all analyzed specimens were fully developed adults, no correction for ontogenetic growth stage was required. Quantitative thresholds for ratio characters are dimensionless, whereas thresholds for absolute distances require compatible units and anatomical definitions. Character definitions and analytical roles are provided in Table S1. The historical acquisition records retained the final character measurements but not replicate landmark placements by the same or different observers; within- and between-observer landmark-acquisition repeatability therefore cannot be estimated retrospectively from the archived data. This limitation concerns validation of the measurement-acquisition step rather than the definition of the taxonomic predictor variables.

Twenty characters (X20–X35, X49, X50, X70, and X71) could be acquired without spreading the wings and were therefore eligible for non-destructive predictive modeling. Here, “non-destructive” means that specimens were not killed, dissected, chemically treated, or permanently altered for character acquisition. Living individuals underwent only brief capture, observation, positioning, and photography before immediate release; no injury or observable adverse effect occurred. X35 and X71 are reciprocal ratios and were retained initially so that their redundancy could be evaluated inside the resampling procedure; coefficients or importance values for these variables were not interpreted as independent biological effects.

### 2.3. Exploratory Numerical Analysis

**Figure 2:**
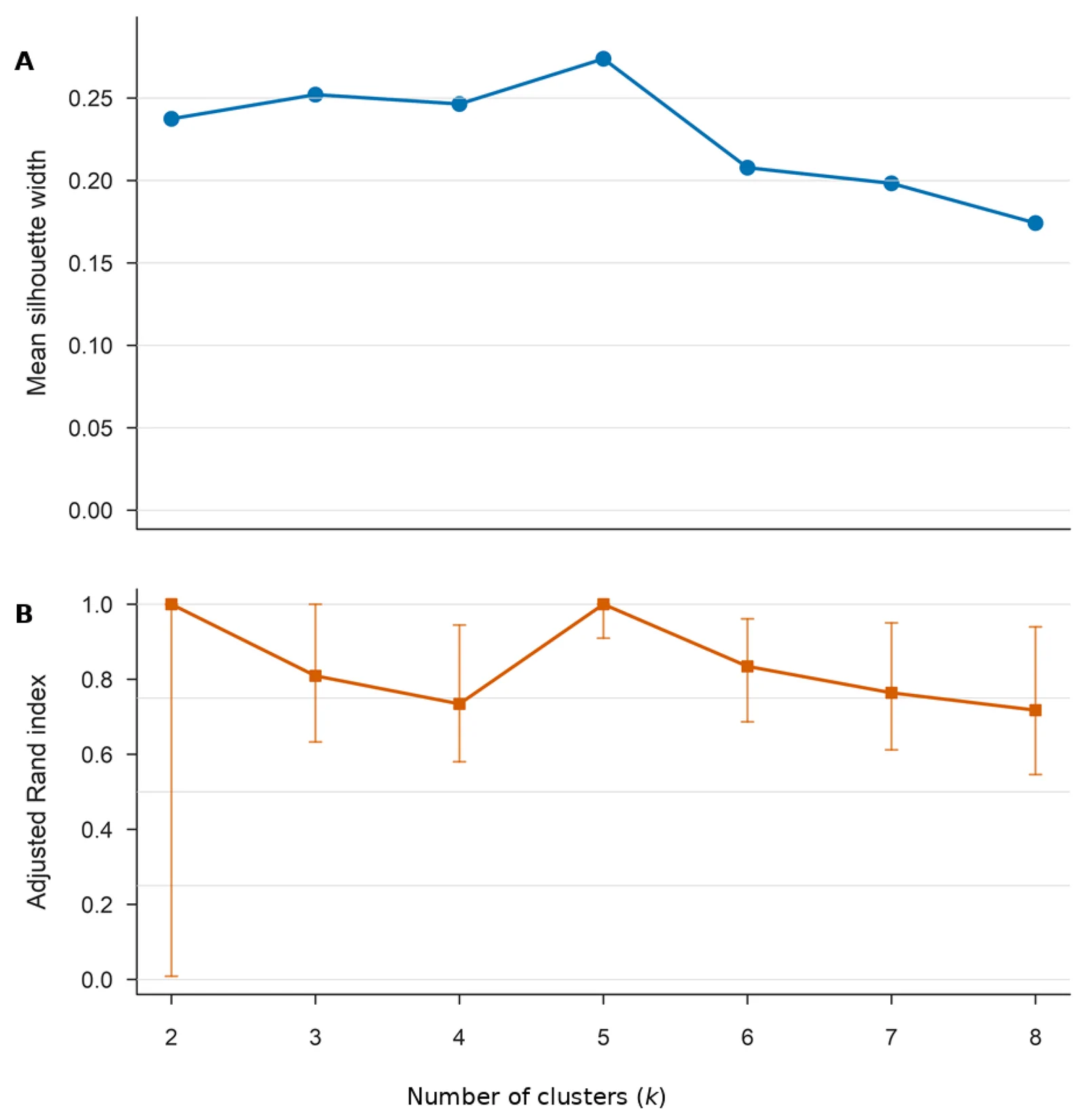
Selection and perturbation stability of morphological groups. (**A**) Mean silhouette width for PAM solutions with *k* = 2–8. (**B**) Median adjusted Rand index and 2.5–97.5% perturbation interval from 200 iterations retaining 80% of specimens and 80% of characters. The display describes clustering stability and does not delimit species.

The complete 71-character development matrix was analyzed without using species labels to discover broad phenetic structure. Continuous characters were standardized before Euclidean analyses, and Gower distances were retained as a sensitivity analysis consistent with the historical numerical-taxonomy workflow. UPGMA, PAM, and seriate heat maps supplied complementary views of grouping. Cluster number was evaluated by mean silhouette width, and robustness was assessed by 200 perturbations that independently retained 80% of specimens and 80% of characters; adjusted Rand agreement quantified recovery of the full-data partition (Figure 2; Table S5). This resampling asks whether the observed grouping is stable to the available specimen and character composition. It does not prove species status, phylogeny, or absence of gene flow.

### 2.4. Nested Predictive Validation

Analyses were performed in R 4.3.3 using nnet 7.3-19 (multinom), MASS 7.3-60 (lda), class 7.3-22, cluster 2.1.6 (pam, silhouette), and rpart 4.1-23. RDA was implemented from Friedman’s covariance-mixing and diagonal-shrinkage formulation [37] using base R matrix operations so that both tuning parameters remained explicit in the archived code. Random seed 20260718 was set before resampling. Generalization performance was estimated using 20 repeats of stratified five-fold outer cross-validation. Within every outer training set, four-fold inner cross-validation maximized balanced accuracy while selecting the number of retained characters (6, 10, 16, or 20) and weight decay (0.01, 0.1, or 1). Ties favored fewer characters and then stronger regularization. Characters were ranked independently inside each inner-training fold by between-class F statistic. Ranking was predictive screening, not a test of taxonomic significance.

Character selection was deliberately separated into two levels. In the present study, the 20 characters measurable without spreading the wings formed a prespecified practical candidate pool before predictive evaluation. Within each outer training partition, the inner procedure ranked candidates by between-class F statistic and selected the retained feature count from 6, 10, 16, or 20, so the retained set could vary among training partitions without using the outer test fold. In applications to other taxa, the candidate pool may instead be reduced a priori by a taxonomist using previous descriptions, revisionary literature, known diagnostic characters, ease of observation or measurement, non-destructive accessibility, and specimen-preservation constraints. Such expert curation should be fixed before held-out evaluation; if selection is informed by the current labeled data, it should be treated as model selection and repeated within resampling. This hybrid design allows taxonomic experience to determine what is biologically and operationally worth measuring while allowing validation to determine which retained set is actually predictive.

**Figure 3:**
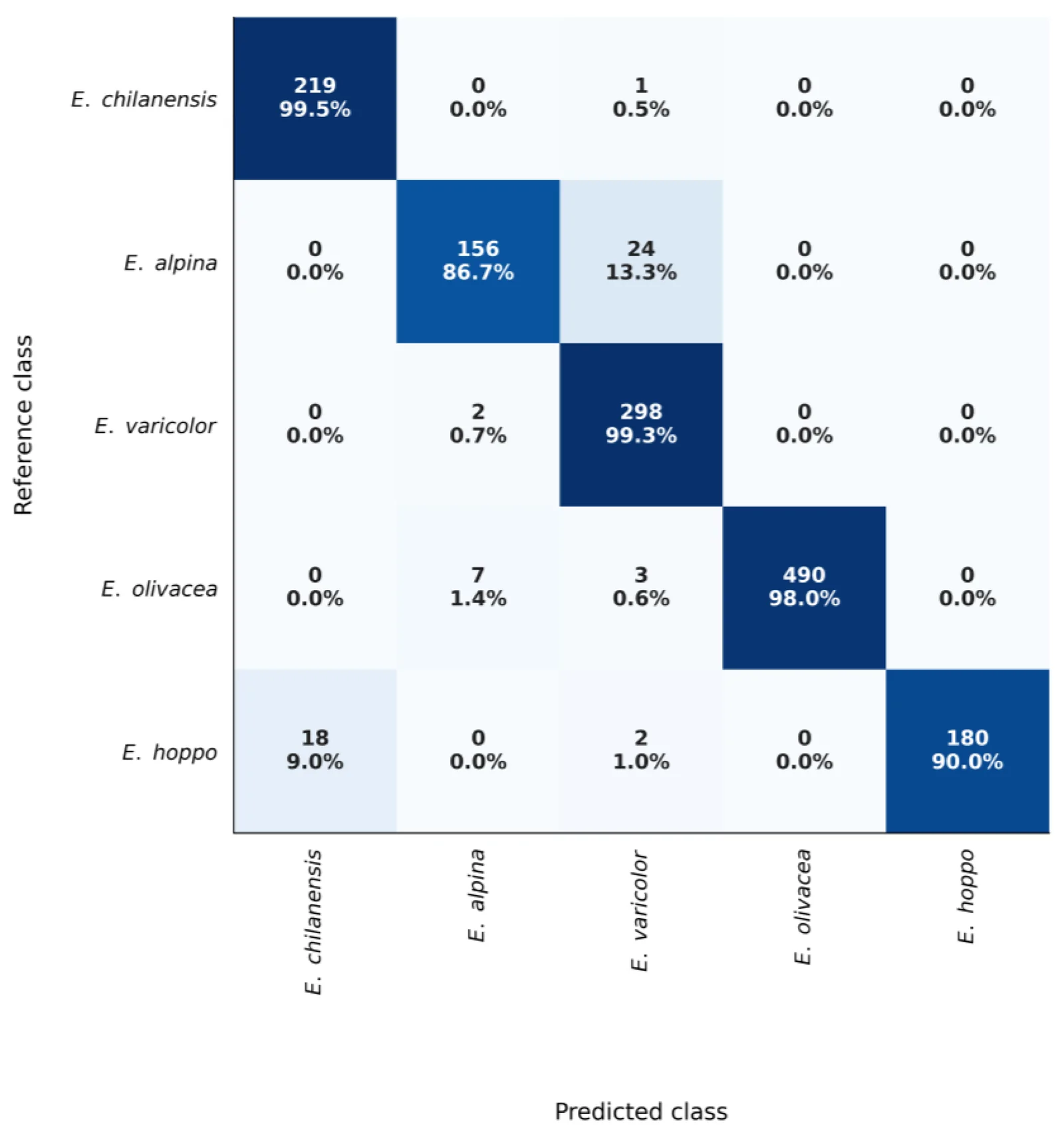
Confusion matrix from 20 × five-fold nested cross-validation. Rows are named operational reference classes, and columns are out-of-sample predictions. Counts aggregate repeated predictions and percentages are row-normalized recalls. Scientific names are italicized, and the x-axis title has been separated from the rotated class labels. Blue shading represents cell magnitude, with darker blue indicating larger counts and row-normalized recall values.

Multinomial log-linear models with weight decay served as the multiclass classifier. Five regularized binomial log-linear models, each contrasting one class against the other four, served as one-versus-rest classifiers. Centering, scaling, ranking, feature-number selection, regularization selection, and fitting were completed without access to the corresponding outer test fold. All compared classifiers used the identical stored outer-fold assignments. The resulting 1400 repeated out-of-fold predictions from 70 biological specimens were pooled to calculate overall accuracy, balanced accuracy, class-specific recall, and the confusion matrix (Figure 3; Tables S2 and S4). Because 20 characters were selected in most outer models, an otherwise identical multinomial model using all 20 characters and weight decay 1 was evaluated as a prespecified simplicity baseline.

### 2.5. Calibration and Benchmark Models

Calibration was assessed internally from outer-fold predictions without refitting on test observations. We calculated multiclass Brier score, mean one-versus-rest Brier score, and top-label expected calibration error (ECE) in 10 equal-width confidence bins. A sensitivity analysis used five equal-width and five equal-frequency bins and recorded both prediction counts and unique contributing specimens. Multinomial and LDA scores were fitted posterior scores, using empirical class priors from each training partition. RDA scores were obtained by exponentiating and row-normalizing regularized Gaussian discriminant functions with the same empirical priors. Nearest-centroid scores were obtained as exp(−d^2^/2), followed by row normalization. For tuned k-nearest neighbors, scores were class proportions among the selected neighbors; distance-weighted voting and factor-level order resolved exact ties deterministically. Tree scores were terminal-node class proportions. These quantities permit Brier-score comparison and calibration assessment but are internally derived model scores, not externally validated probabilities. Reliability diagrams display observed accuracy against mean confidence (Tables S11, S12 and S21).

Seven comparators were evaluated on the identical outer folds: multinomial regression using all 20 characters, multinomial regression restricted to 17 dimensionless ratios, LDA, RDA, nearest-centroid, tuned *k*-nearest neighbors, and the pruned classification tree. RDA covariance mixing and diagonal shrinkage, and the neighbor count, were selected by four-fold cross-validation restricted to each outer training partition. Comparisons included accuracy, balanced accuracy, Brier score, ECE, fitting time, and a descriptive analysis near 82% coverage (Tables S14–S19). Matched-coverage cutoffs were chosen from pooled out-of-fold scores and are descriptive, not independent operating thresholds.

Uncertainty was estimated with 2000 paired cluster-bootstrap replicates at the biological-specimen level. Specimen identifiers, rather than 1400 repeated predictions, were sampled with replacement; all 20 predictions for a sampled specimen remained together. The same resampled specimens were used across models, permitting paired intervals for model-to-model differences. We bootstrapped accuracy, balanced accuracy, Brier scores, ECE, coverage, conditional accuracy, and agreement-rule performance. As a sensitivity analysis, each metric was also calculated within each of the 20 repeats before summarizing its between-repeat distribution (Tables S16–S19). Percentile intervals are 95% confidence intervals.

### 2.6. Selective Classification

**Figure 4:**
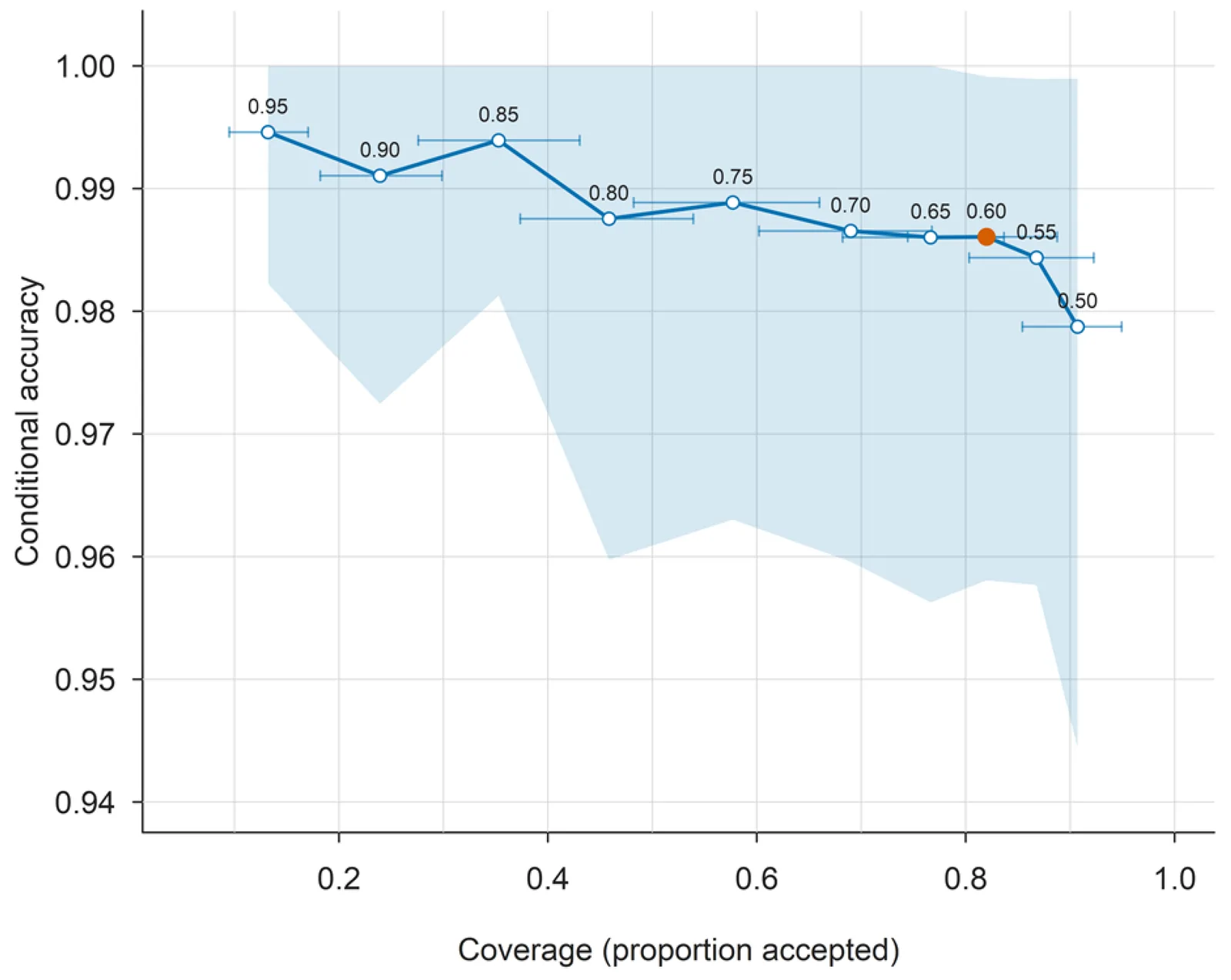
Accuracy–coverage trade-off for the agreement rule. Points show thresholds from 0.50 to 0.95; ribbons show specimen-cluster bootstrap 95% intervals. The orange highlighted circle marks the illustrative working threshold t = 0.60. The y-axis begins at 0.94 to display the full observed conditional-accuracy range without implying large absolute differences; t = 0.60 was not independently prespecified.

The historical agreement requirement was formalized as a reject-option classifier. For acceptance at threshold *t*, the one-versus-rest score for the class selected by the multiclass model had to be ≥*t*, and the largest score from the four competing models had to be <1 − *t*. These outputs are model scores requiring empirical calibration; they are not assumed to be mutually calibrated posterior probabilities. All other predictions were rejected. Thresholds from 0.50 to 0.95 were used to calculate coverage, rejection rate, conditional accuracy among accepted predictions, and the proportion simultaneously accepted and correct (Figure 4; Table S3). The value 0.60 was selected after inspecting the accuracy–coverage curve and is reported as an illustrative working threshold, not as a prespecified or independently optimized threshold.

### 2.7. Provisional Quantitative Screening Tree

To accelerate construction of an interpretable identification key, we fitted CART to the 20 external characters. The tree learns the order of characters and quantitative thresholds from labeled reference specimens and can be regenerated when the reference library changes. Because each split independently chooses the character and threshold that best separates the remaining classes, CART also performs branch-specific character selection and can expose a compact subset of characters useful for a practical key. The starting candidate pool can be broad or can be prespecified by a taxonomist when particular characters are preferred because they are easy to observe, non-destructive, or already established in the literature. Tree complexity (cp = 0, 0.001, 0.003, 0.01, 0.03, or 0.05) and minimum split size (4, 6, or 8 specimens) were selected by four-fold inner cross-validation within each partition of the same 20 repeats of five-fold outer resampling; maximum depth was six. Accuracy and balanced accuracy were calculated only from outer-fold predictions. A final pruned tree fitted to all 70 specimens was exported as a provisional, automatically generated first draft of a quantitative key. Taxonomists must still inspect threshold-adjacent specimens, add qualitative characters where useful, and validate the final key independently.

### 2.8. Open-Set Taxonomic Projection

The final classifiers were fitted to all 70 development specimens with the 20 non-destructive characters and weight decay 1, the modal choice in nested resampling. Projection specimens were first evaluated at t = 0.60. Characters were standardized using development-set means and standard deviations. A pooled within-class covariance matrix S was regularized as S_r_ = (1 − λ)S + λ diag(S), with λ = 0.5 fixed before projection. This prespecified mid-range shrinkage was retained to stabilize inversion in the small-sample projection analysis and to avoid tuning a rejection rule on the heterogeneous projection set; it is a design choice rather than an estimated optimum. Squared Mahalanobis distance from a specimen to its predicted-class centroid was compared with a single pooled 95th percentile (type-8 quantile) of leave-one-out distances of development specimens from their own leave-one-out class centroids; no class-specific cutoff was used. Acceptance required classifier agreement and a distance below this cutoff. PCA was calculated from the centered and scaled 20-character development matrix and used only for two-dimensional display.

### 2.9. Leave-One-Species-Out Unknown-Class Validation

Open-set performance was evaluated separately from the heterogeneous projection specimens. Each reference species was omitted in turn and designated the positive “unknown” class. In each of 20 repeats, the remaining four classes were partitioned into five stratified folds. The multinomial model, empirical class priors, class centroids, pooled covariance, and single pooled 95th-percentile distance cutoff were recalculated independently from each training partition. Each represented specimen was evaluated once in its held-out fold. Each omitted specimen was initially evaluated by all five-fold-specific models; to prevent five-fold over-weighting, its five distance-to-cutoff ratios were averaged to one specimen-repeat novelty score, which was rejected when the mean ratio exceeded 1. The mean was prespecified because it retains the magnitude of evidence from every fold model on the common cutoff-normalized scale, whereas majority voting discards magnitude. Because this choice is not unique, a sensitivity analysis repeated unknown rejection using the median ratio and a majority rule requiring rejection by at least three of the five-fold models. A once-per-repeat model refitted to all four retained species was not mixed into the primary analysis because it would use a different training-set size from the held-out known-specimen evaluation; such a model is better treated as a future deployment model rather than a directly paired validation rule. Each known specimen likewise contributed one specimen-repeat score. Thus, known and unknown specimens had equal biological weight: 20 decisions per specimen across repeats. The omitted/known specimen counts were 11/59, 9/61, 15/55, 25/45 and 10/60 for *E. chilanensis*, *E. alpina*, *E. varicolor*, *E. olivacea* and *E. hoppo*, respectively.

**Figure 5:**
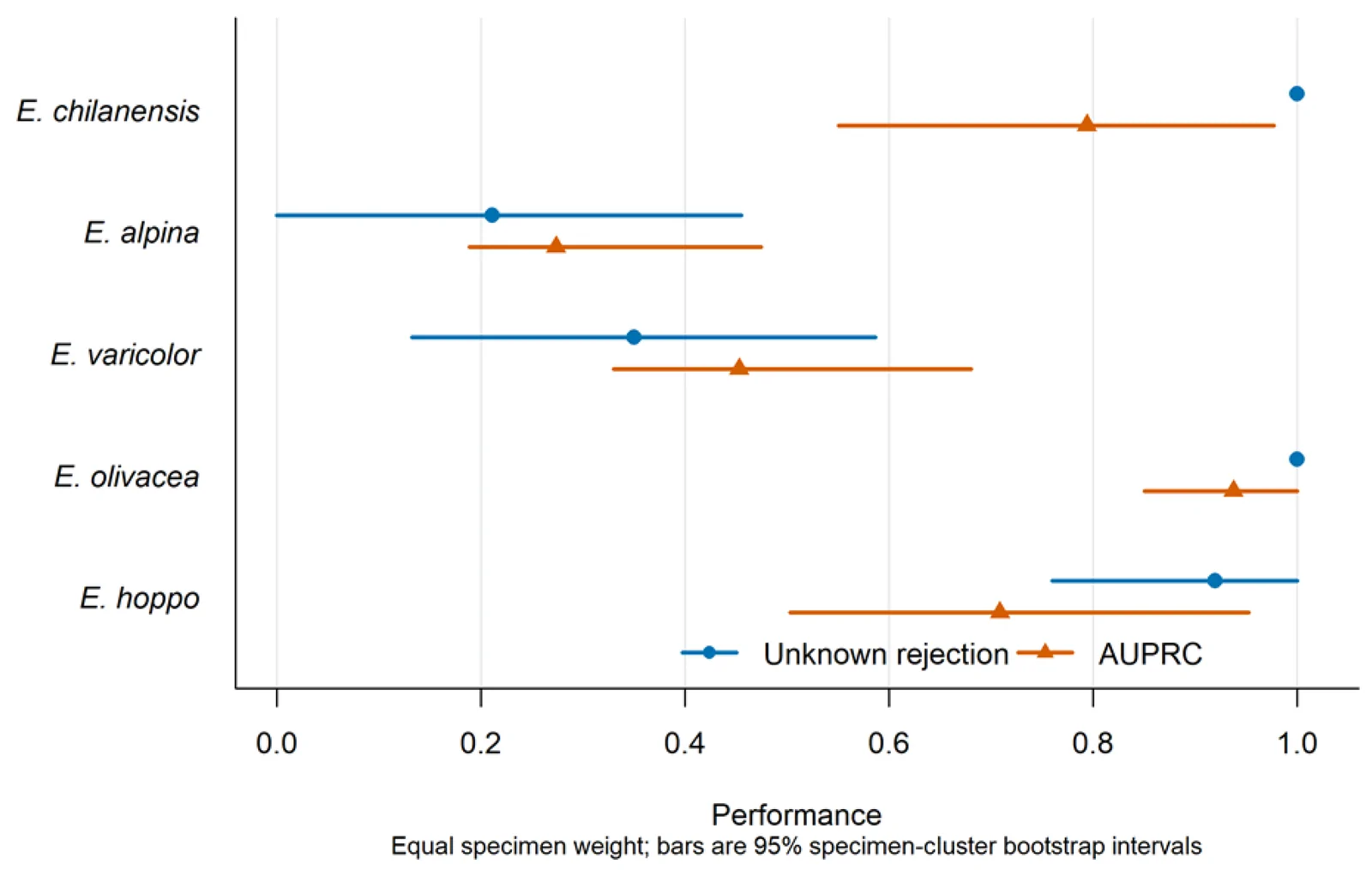
Leave-one-species-out evaluation of the distance-based unknown-class screen. Points and intervals show equal-biological-weight estimates and empirical specimen-cluster bootstrap intervals for unknown-class rejection and AUPRC when each named species was omitted completely from model development. Performance varies markedly among omitted species.

The normalized novelty score for AUROC and AUPRC was the squared regularized Mahalanobis distance divided by its fold-specific cutoff; larger values indicated greater evidence for “unknown.” We report unique specimen counts, specimen-repeat counts, effective unknown prevalence, the prevalence-based no-skill AUPRC, unknown rejection, false acceptance, known false rejection, AUROC, AUPRC, empirical open-set accuracy, and balanced open-set accuracy. Empirical open-set accuracy weights specimens at their observed prevalence; balanced open-set accuracy is the mean of accepted-and-correct known rate and unknown rejection, giving known and unknown status equal importance. Five hundred stratified specimen-cluster bootstrap replicates resampled known and unknown biological specimens separately while retaining all 20 specimen-repeat decisions for each sampled specimen. This design quantifies omitted-species detection within the current measurement domain, not unknown genera or new imaging domains (Figure 5; Tables S13, S20 and S22).

### 2.10. Taxonomic Interpretation

Predictive membership, morphological diagnosis, species delimitation, phylogenetic inference, and nomenclatural action were treated as distinct steps. Models assessed similarity to operational reference hypotheses and the reliability of identification decisions. Synonymy and species status additionally require name-bearing types, original descriptions, priority, diagnostic morphology, and relevant geographic, acoustic, ecological, and genetic evidence. As an optional descriptive aid in prospective species descriptions based on a series, the PAM medoid may be reported as a reproducible measure of morphological representativeness and used to inform, but not determine, a taxonomist’s selection of a candidate holotype. The algorithm does not designate the type and does not override the International Code of Zoological Nomenclature or curatorial judgment.

## 3. Results

### 3.1. Data Audit

The 70-specimen development matrix contained no missing values or duplicate specimen identifiers. Reference-class sizes were 11, 9, 15, 25, and 10. Several characters were strongly correlated; X35 and X71 were nearly perfectly inversely correlated, as expected from their definitions. A later 2019 workbook contained the same specimen order and values as the high-precision 2016 development matrix, except that X1 had been rounded to two decimal places. Analyses therefore used the unrounded values.

### 3.2. Out-of-Sample Classification

Across PAM solutions from two to eight groups, the five-group solution had the highest mean silhouette width (0.274). Under 200 perturbations that independently retained 80% of specimens and 80% of characters, its median adjusted Rand agreement with the full-data solution was 1.000 (95% empirical interval 0.910–1.000; Figure 2). Solutions with three, four, and six to eight groups had lower silhouette widths and lower median stability. The two-group solution also had a median adjusted Rand index of 1.000 but a much lower 2.5th percentile (0.009), indicating occasional collapse under perturbation. These results describe structure in the measured morphospace; they do not assign species rank.

Nested resampling produced 1400 repeated out-of-fold predictions from 70 biological specimens, which remained the independent clusters for uncertainty estimation. Feature-screened multinomial accuracy was 95.93% (specimen-cluster bootstrap 95% CI 91.36–99.48%) and balanced accuracy was 94.71% (87.90–99.38%). Recalls for *E. chilanensis*, *E. alpina*, *E. varicolor*, *E. olivacea*, and *E. hoppo* were 99.5%, 86.7%, 99.3%, 98.0%, and 90.0%, respectively (Figure 3). Errors concentrated between *E. alpina* and *E. varicolor* and between *E. hoppo* and *E. chilanensis*. These values quantify the prediction of unseen morphological measurements relative to the defined reference classes.

All 20 candidate characters were selected in 94 of 100 outer models. The all-character multinomial model had the highest closed-set accuracy (96.57%, 95% CI 92.21–99.93%) and balanced accuracy (95.41%, 88.60–99.90%), followed by the feature-screened multinomial model (95.93%; 94.71%) and RDA (95.79%; 94.24%). The paired accuracy difference between feature-screened and all-character multinomial models was −0.64 percentage points (95% CI −1.29 to −0.14), confirming that screening did not improve prediction. This negative result is informative for the character-selection workflow: the starting 20-character pool was already compact and broadly useful, and nested selection correctly did not force parsimony when the held-out data did not support it. RDA traded a small, uncertain accuracy difference (−0.79 points, −2.33 to 0.36) for a lower Brier score (difference −0.033, −0.055 to −0.010) and lower ECE (−0.103, −0.124 to −0.075). The ratios-only model achieved 92.93% accuracy, showing that dimensionless shape information remained substantial but did not equal the mixed distance-and-ratio set. Full model estimates, cluster intervals, paired differences, and repeat-level sensitivity appear in Tables S14–S19.

### 3.3. Selective-Classification Performance

At *t* = 0.50, 90.71% of predictions were accepted, and conditional accuracy was 97.87%. At the illustrative threshold *t* = 0.60, agreement-rule coverage was 82.00% (specimen-cluster 95% CI 74.64–88.55%) and conditional accuracy was 98.61% (96.16–99.92%; Figure 4). More conservative thresholds increased reliability while sharply reducing coverage. At descriptively matched 82% coverage, conditional accuracy was 99.83% for the all-character multinomial score, 99.39% for RDA, and 98.17% for the feature-screened multinomial score. The agreement rule therefore supplies a transparent disagreement flag but is not the principal performance gain and did not outperform simpler confidence rejection (Tables S15–S19).

### 3.4. Probability Calibration

**Figure 6:**
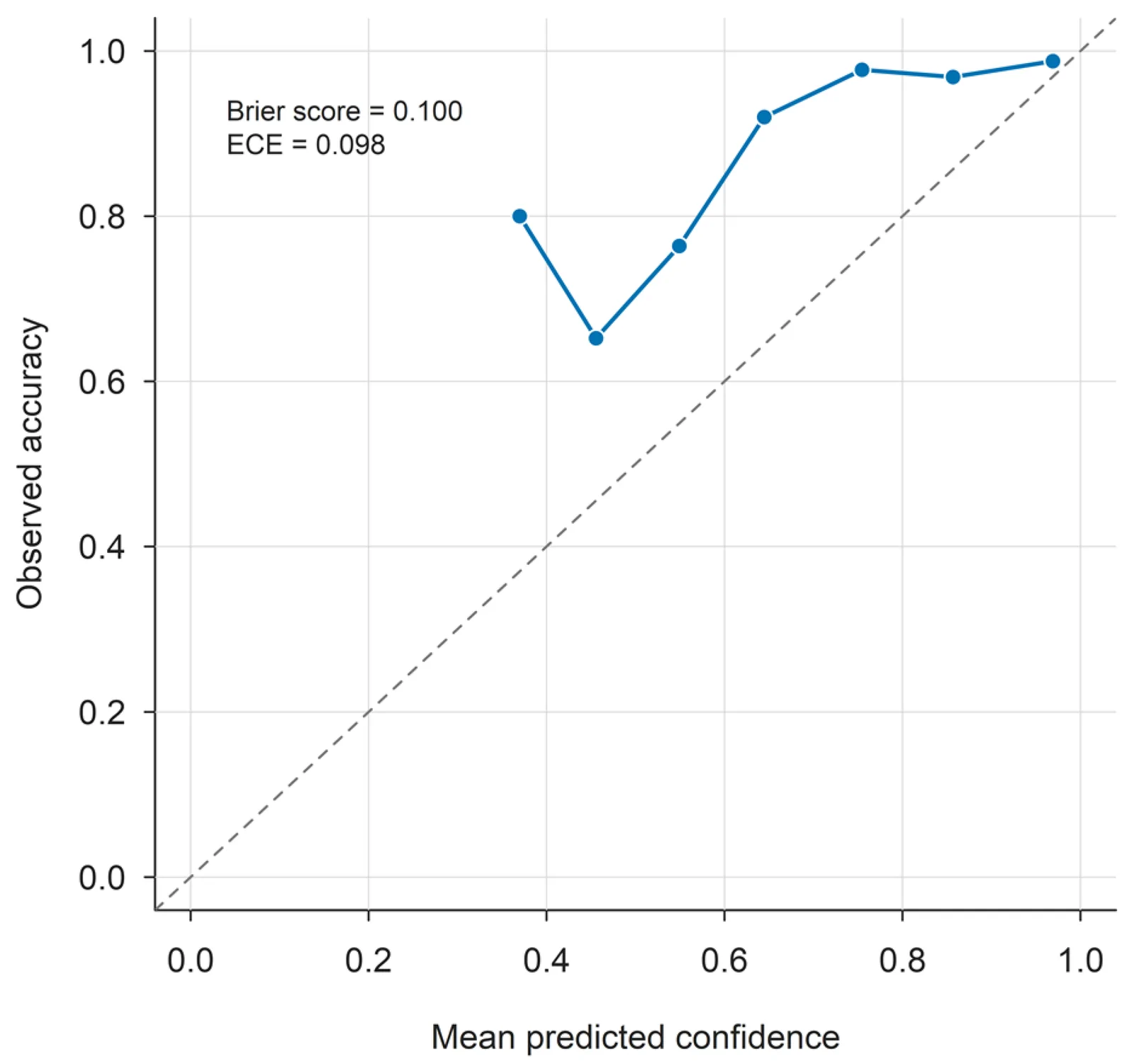
Out-of-fold reliability of the multiclass classifier. Observed accuracy is plotted against mean predicted confidence in 10 equal-width bins; the diagonal denotes perfect calibration. Brier score and expected calibration error summarize deviations.

For the feature-screened multinomial component, the multiclass Brier score was 0.100 (specimen-cluster 95% CI 0.054–0.165), mean one-versus-rest Brier score was 0.031 (0.022–0.044), and 10-bin top-label ECE was 0.098 (0.061–0.136; Figure 6; Tables S11, S12, S16 and S21). Five-bin sensitivity analyses led to the same qualitative conclusion. In five equal-width bins, only one unique specimen contributed to the lowest occupied confidence region; equal-frequency bins contained 32–54 unique specimens, but the same specimens could contribute to multiple bins across repeats. Calibration is therefore internal and imprecise in sparse low-confidence regions. RDA showed better internal calibration, but the small specimen count precludes treating any score as a universally calibrated probability. Thresholds are decision settings, not taxonomic posterior probabilities.

### 3.5. Leave-One-Species-Out Open-Set Performance

After aggregation to one decision per specimen per repeat, unknown-class rejection remained strongly taxon-dependent (Figure 5; Tables S13, S20 and S22). Rejection was 100% for *E. chilanensis* (empirical specimen-cluster bootstrap interval 100–100%), 100% for *E. olivacea* (100–100%) and 92.0% for *E. hoppo* (76.0–100%). These empirical intervals describe stability under resampling of the finite observed specimens; a 100–100% interval does not imply that the rejection probability for future specimens is necessarily 100%. By contrast, rejection was 21.1% for *E. alpina* (0–45.5%; false acceptance 78.9%) and 35.0% for *E. varicolor* (13.2–58.7%; false acceptance 65.0%). Corrected AUPRC values were 0.794, 0.938, 0.709, 0.274 and 0.453 for *E. chilanensis*, *E. olivacea*, *E. hoppo*, *E. alpina* and *E. varicolor*, respectively; their no-skill baselines were the effective unknown prevalences 0.157, 0.357, 0.143, 0.129 and 0.214. Balanced open-set accuracies were 0.933, 0.938, 0.892, 0.556 and 0.621, respectively. The wide intervals reflect only 9–25 independent unknown specimens. Equal biological weighting substantially reduced prevalence-sensitive AUPRC estimates but preserved the central conclusion: reference-library absence is detectable when an omitted taxon is separated in measured morphospace and may be invisible when it overlaps represented species. Aggregation-rule sensitivity supported the same conclusion. Mean, median and majority-vote rejection rates were respectively 21.1%, 15.0% and 15.0% for *E. alpina*; 100%, 99.5% and 99.5% for *E. chilanensis*; 92.0%, 92.5% and 92.5% for *E. hoppo*; 100% under all three rules for *E. olivacea*; and 35.0%, 30.0% and 30.0% for *E. varicolor* (Table S22). Median and majority voting were identical in point estimates because five-fold votes yield an odd decision count; modest mean-versus-median differences did not alter the species ranking or inference.

### 3.6. Projection of Taxonomically Important Material

**Figure 7:**
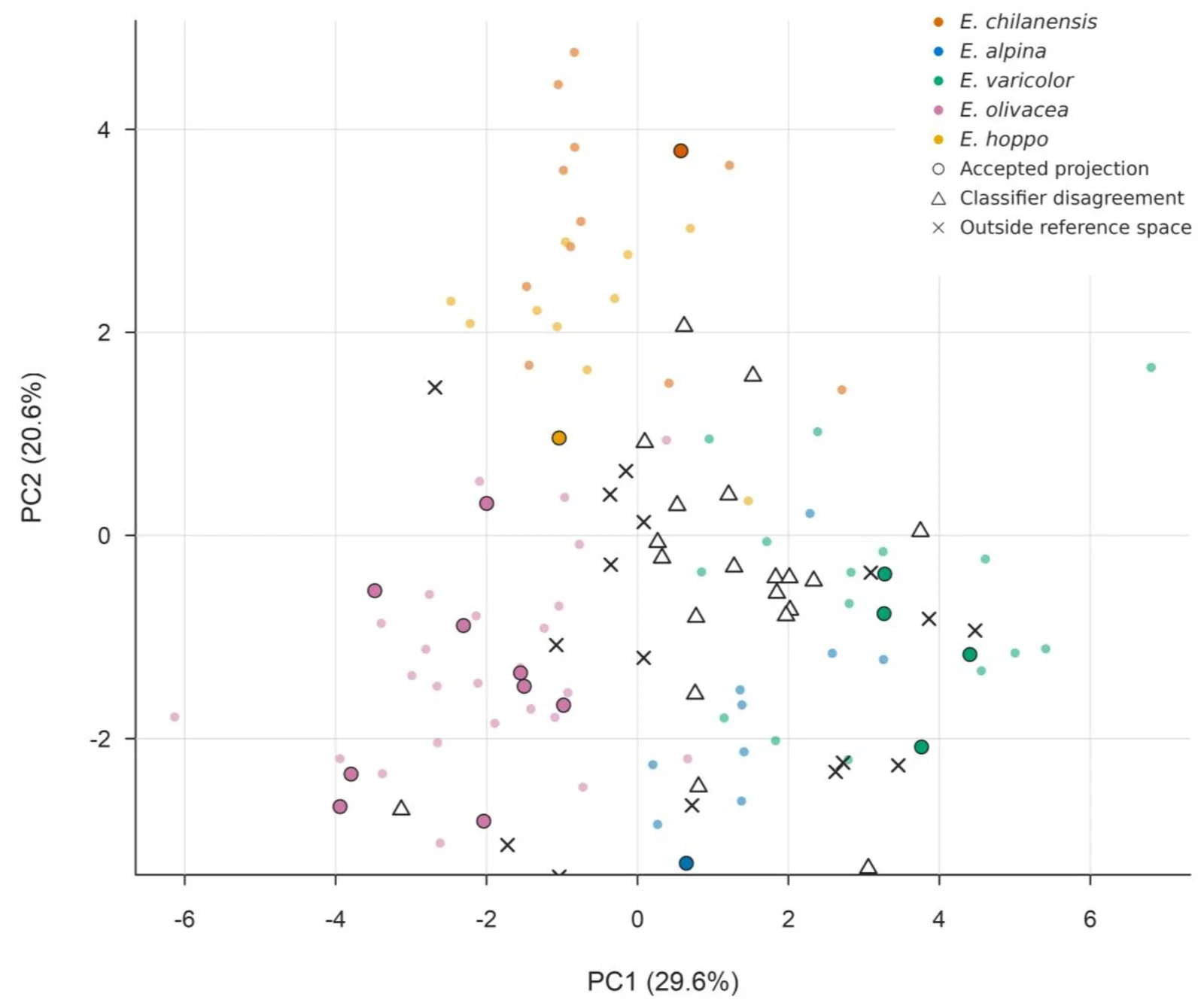
PCA display of development and taxonomic-projection specimens. Filled translucent circles are development specimens colored by named reference class. Projected specimens are distinguished by outlined circles (accepted), triangles (classifier disagreement), and crosses (distance rejection). PCA is used only for visualization and is not evidence of open-set performance.

Sixteen of 55 non-duplicated projection specimens satisfied classifier agreement and the distance criterion; 20 were rejected because component classifiers disagreed and 19 because they exceeded the distance cutoff (Figure 7). Because the leave-one-species-out analysis showed high false acceptance for some omitted species, these projection outcomes are exploratory similarity assessments rather than independent open-set validation. Results are reported with species names in Tables S6 and S7; no synonymy, identification of an unrepresented taxon, or nomenclatural conclusion is inferred from an accepted projection.

### 3.7. Performance of the Quantitative Screening Tree

**Figure 8:**
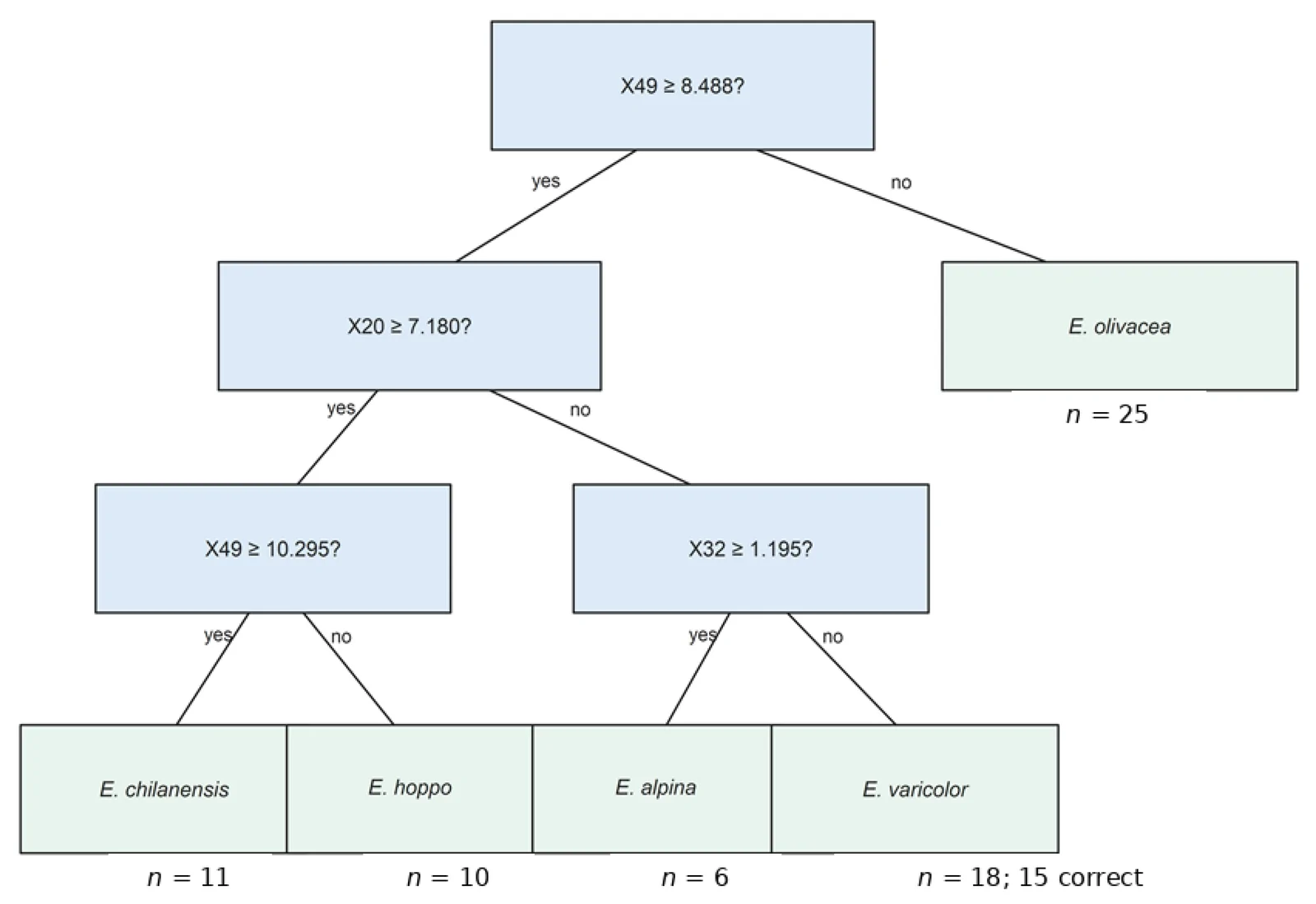
Provisional quantitative screening tree for the five operational *Euterpnosia* reference classes. Internal nodes show measured thresholds; terminal nodes show the first-pass classification and development-sample count. The tree is an interpretable screening aid rather than an operational taxonomic key, open-set model, or species-delimitation method. Blue boxes indicate internal decision nodes, and green boxes indicate terminal classification nodes. In the terminal nodes, *n* denotes the development-sample count.

Dedicated nested tuning yielded 80.57% accuracy and 76.61% balanced accuracy (Tables S9 and S10); common benchmark folds gave 82.00% and 78.02%, respectively (Table S14). The final tree contained five terminal leaves and selected only X49, X20, and X32 from the 20-character candidate pool (Figure 8). This compact three-character solution illustrates a different objective from the higher-performing multinomial classifier: CART sacrifices some predictive accuracy in exchange for a short, transparent sequence of branch-specific diagnostic measurements. One terminal leaf contained classification errors, and X49 is an absolute abdominal distance and therefore may encode diagnostically useful adult-size information in addition to proportional morphology. The result demonstrates that CART can rapidly produce an interpretable first draft of a quantitative key and an empirically prioritized character subset, but expert revision and independent validation are required before operational use.

## 4. Discussion

### 4.1. From Numerical Taxonomy to Classifier Modeling

The contribution of the workflow is the connection among successive taxonomic tasks rather than a contest for the highest accuracy. Unsupervised numerical analysis first summarizes structure across 71 characters without requiring a prior explanation for every observed difference. Perturbation stability then asks whether that structure persists when available specimens and characters change. Supervised models subsequently test diagnosability relative to operationally labeled references; omission experiments challenge library completeness; and a classification tree converts multivariate results into an inspectable key draft. Because the models consume defined anatomical variables rather than raw pixels, taxonomists can combine automated calculation with characters already emphasized in descriptions and revisions. Once landmarks or direct measurements have been recorded, distance and ratio calculation, screening, fitting, projection, and tree generation can be automated, reducing repetitive work while preserving an auditable connection to morphology.

No classifier dominated every criterion. The all-character multinomial model maximized closed-set accuracy, RDA yielded better internal calibration metrics, and the compound agreement rule supplied an auditable reason for abstention without outperforming simpler confidence rejection at matched coverage. This confirms that the workflow is algorithm-agnostic: unsupervised and supervised methods may be substituted when justified, provided that feature selection, tuning, validation, rejection, and taxonomic interpretation retain the same separation of roles.

### 4.2. Character Selection as a Bridge to Traditional Taxonomy

For practicing taxonomists, character selection may be one of the most useful outputs of the workflow. Much traditional revisionary work is devoted to discovering characters that are reliable, easy to observe, non-destructive, and sufficiently discriminating either alone or in combination. The workflow formalizes that search without displacing expert judgment. A broad character matrix can be retained for unsupervised discovery, whereas supervised identification can begin from an expert-defined candidate pool and then use training-only screening or regularization to rank and retain predictive characters. CART adds a complementary layer by selecting different characters at different branches. The resulting ranked or selected variables can be used not only inside a classifier but also as empirical leads for diagnoses, quantitative keys, future descriptions, and decisions about which characters deserve additional sampling. This extends the variable-selection logic used in earlier work on classifier modeling [21] and the reliability ranking of numeric diagnostic characters in *Euterpnosia* [22].

The present data also show why character selection should be treated as an evidence-driven option rather than an assumption that fewer variables must always be better. Nested screening retained all 20 candidate characters in 94 of 100 outer models and did not improve multinomial accuracy, indicating that the prespecified 20-character pool was already compact and information-rich. By contrast, the final CART key used only X49, X20, and X32, but with lower predictive accuracy. Thus, the preferred character set depends on the taxonomic task: a richer set may be appropriate when maximizing classifier performance, whereas a smaller set of readily measured characters may be preferable for a field or collection key. In larger character libraries, validated selection can also reduce measurement and model-fitting effort; in the present small data set, however, computational time itself was not a limiting factor. Importantly, statistical selection identifies candidate diagnostic utility relative to the reference data; the biological meaning, taxonomic reliability, and suitability of those characters still require expert examination.

### 4.3. Reference-Library Overlap Is the Critical Failure Mode

The leave-one-species-out experiment supplies the broadest biological inference. Open-set recognition is often discussed as though “unknown” were an intrinsic detectable state. It is not. A specimen is unknown relative to a library, whereas rejection depends on its position relative to represented morphospace. *E. chilanensis*, *E. hoppo*, and *E. olivacea* produced strong novelty signals when omitted; *E. alpina* and *E. varicolor* were usually absorbed by retained classes. High closed-set accuracy can therefore coexist with poor protection against a biologically plausible missing species.

For systematic research, this changes how automated identifications should be interpreted. Acceptance means that a specimen resembles one represented hypothesis under the measured characters; it does not demonstrate reference completeness. Rejection identifies insufficient support for an assignment but does not establish a new species. Reliable deployment requires deliberate “challenge taxa” chosen for morphological proximity, not only random withholding from well-separated classes. This principle applies beyond cicadas to morphometric keys, barcode reference systems, and image classifiers whenever the taxonomic library is incomplete.

### 4.4. Phenetic Evidence Within Integrative and Iterative Taxonomy

Phenetic classification and phylogenetic systematics address different questions. The former summarizes resemblance among observed organisms; the latter proposes historical relationships and causal explanations. Accordingly, we explicitly treat species as hypotheses rather than statistical classes [9,10]. A stable morphological cluster can motivate a descriptive or diagnostic hypothesis, but it has no automatic species rank and does not indicate monophyly. A classifier can test diagnosability relative to named references, and an open-set procedure can identify unsupported projections, but formal delimitation must integrate types, morphology, calling song, geography, ecology, genetics where available, and nomenclature. Thus, the present analysis is phenetic in its pattern-discovery stage and taxonomic in its diagnostic application; it is not a phylogenetic reconstruction.

The history of *E. chilanensis* illustrates this iterative relationship but is not independent prospective validation. The quantitative work and subsequent description shared a research program and an author, and the present model uses *E. chilanensis* as an already labeled class. Chen et al. (2021) is therefore cited as historical context showing compatibility with a later conventional description, not as proof that the present model discovered an unknown species. A genuine reconstruction would require freezing the pre-description data and labels and withholding all information that encoded the later taxonomic hypothesis.

### 4.5. Automated Quantitative Key Construction

Traditional dichotomous keys remain indispensable because their decisions are inspectable and usable without statistical software [8]. Constructing them becomes laborious as species and candidate characters increase, because the author must choose an ordering, translate continuous variation into states, and revisit earlier couplets whenever new taxa are added. CART automates the first draft of this work by selecting branch-specific characters and thresholds from the training matrix. The resulting tree can be regenerated after library expansion, tested on specimens withheld from tree construction, and distributed as a printed or interactive quantitative key. This is a practical bridge from multivariate modeling back to conventional taxonomic communication.

The present tree is not a finished replacement for an expert key: hard splits discard information near thresholds, accuracy was lower than that of multivariate models, and the tree has no intrinsic unknown-class mechanism. Its value lies in rapid, objective key drafting. The tree supplies a human-readable first pass, probabilistic models provide confirmation and abstention, and the taxonomist reviews thresholds, adds qualitative characters where needed, and retains responsibility for diagnosis and nomenclature. Even where the final key is edited, the algorithm substantially reduces the search burden and makes the empirical error of the initial key explicit.

### 4.6. Flexible Specimen-, Live-Individual-, and Image-Enabled Taxonomy

The framework is useful both within conventional collection-based taxonomy and when dissection or destructive sampling is undesirable, because the selected 20 characters can be measured without spreading the wings. Seventeen of these 20 predictive characters are dimensionless ratios among homologous landmark distances. Consequently, most of the predictive character set can be obtained from a suitably oriented image without a physical scale bar: uniform magnification changes both component distances by the same factor and therefore leaves their ratio unchanged. A scale bar or other calibration is needed only when absolute dimensions are to be added, such as body, head, or wing lengths and widths; calibration can therefore expand the available character set but is not essential when the ratio characters already provide sufficient taxonomic discrimination. This acquisition flexibility allows direct examination of specimens and living individuals, image-derived measurements, or a compatible combination of both according to material availability and the biological question. Unlike calling song and male genitalia, these external measurements are technically available from both sexes. This creates a route toward female identification in a group whose traditional taxonomy is male-centered. The present archive cannot quantify female-specific accuracy, so that advantage is methodological capability rather than a completed sex-transfer validation. A targeted female reference series is an immediate and biologically important next test.

The workflow intentionally uses taxonomic morphology, rather than observer identity, camera model, collector, collection date, locality label, or other collection metadata, as predictor information. Such metadata can be valuable for curation, ecology, or biogeography, but they are not morphological characters and are not required to calculate the present classifier. They should nevertheless be distinguished from validation-design information. When prospectively recorded, observer, locality, imaging session or camera, preservation state, and related metadata can serve as grouping or blocking variables for testing whether a fitted classifier transports across operators, populations, or acquisition domains. The historical dataset does not contain these fields in sufficient detail for such blocked validation, so the present results should not be interpreted as an independent demonstration of cross-observer or cross-domain transfer. For ratio-based characters, uniform image magnification cancels out mathematically, reducing dependence on the absolute image scale; this property does not eliminate possible error from landmark placement, specimen positioning, or other acquisition steps. Standardized anatomical definitions and consistent landmark placement therefore remain important for reproducible measurement [38,39,40].

### 4.7. Taxonomic Implications and Limits

The projection results do not provide independent support for synonymy. The *E. elongata* holotype and material historically identified as *E. kotoshoensis* contributed to reference-class construction, so their subsequent fitted or projected positions partly reproduce supplied labels. These specimens remain relevant to type-based comparison, but nomenclatural conclusions require an explicitly independent revision.

The leave-one-species-out results define a sharper limit. Morphologically separated omissions were usually rejected, whereas most omitted *E. alpina* and *E. varicolor* specimens were falsely accepted. Distance rejection can flag some gaps in a reference library, but absence from the library is not identifiable when an unknown overlaps known morphospace. Neither acceptance nor rejection is evidence of species status.

### 4.8. Limitations and Future Validation

The five reference classes comprise 70 unequal and predominantly male specimens collected over seven years. For rare wild organisms, no universal minimum sample size can be specified independently of within-taxon variation and between-taxon separation. Here, the five-group solution remained highly stable under repeated specimen and character perturbation, supporting adequacy for detecting structure in this particular matrix. The 20-character predictive set is dominated by 17 dimensionless ratios, and the ratios-only benchmark still achieved 92.93% accuracy, showing that scale-free relative morphology alone contains substantial diagnostic signal. The three absolute-distance characters provide additional information when calibrated measurements are available, but the classifier does not depend exclusively on absolute size. Because all specimens were fully developed adults, developmental-stage growth was not a source of variation. Static adult size-trait relationships were not separately decomposed, and the present results should not be read as proof that static allometry is absent; absolute adult dimensions may themselves carry diagnostic information for the present classification objective. A second limitation is validation of measurement acquisition and transportability. Replicate landmarking records by the same or different observers were not retained, and the historical metadata are incomplete for blocking by observer, locality, imaging session, camera, or preservation state. Accordingly, the reported performance quantifies prediction from the cleaned and harmonized measurements within the present reference domain, not independent cross-operator or cross-domain transfer. Future prospective validation should record replicate landmarking and relevant blocking metadata, include more female specimens and broader taxonomic and geographic sampling, and incorporate complementary acoustic or molecular evidence where appropriate.

Computational reproducibility and reconstruction of raw measurements must be distinguished. The supplied archive reproduces the statistical stage from the cleaned and harmonized character matrices, including fold assignments, model scores, tuning, bootstrap results, figures, session information, and checksums. The complete workflow was re-executed end-to-end with R 4.3.3 on Windows 11; it completed with exit code 0 and produced 38 regenerated files whose SHA-256 checksums were independently verified. The archive includes the execution record, regenerated outputs, session information, and output-checksum manifest. The original photographs and landmark-coordinate files are not retained in the present project archive and are no longer available from the corresponding author. Most voucher and type material remains in the public institutional collections identified above and may be re-examined, re-photographed, or remeasured with collection approval. Consequently, the archived analysis is computationally reproducible from processed measurements, whereas a complete independent reconstruction of landmark acquisition requires renewed access to the vouchers.

## 5. Conclusions

This study restores the full connection between numerical taxonomy and practical identification: unsupervised morphology discovers and tests the stability of operational groups; classifier modeling selects and validates diagnostic characters and classifies new measurements; abstention and omission tests expose unsupported assignments; taxonomically important material can then be projected for expert interpretation; and CART rapidly generates an auditable first draft of a quantitative key. A further contribution is the explicit separation between broad character discovery and goal-specific character selection. Taxonomists may prespecify candidate characters from literature, experience, accessibility, and preservation constraints, while training-only algorithms can evaluate retained sets and character combinations without leaking information from held-out specimens. The resulting character rankings and subsets can therefore serve both classifier modeling and traditional diagnosis or key development. Within the present *Euterpnosia* reference domain, external morphology supported high out-of-sample accuracy, although nested feature screening did not outperform the already compact 20-character set. The strongest result, nevertheless, was the failure mode: omitted species were rejected only when separated from the represented morphospace. As an optional extension, a medoid-based measure of morphological representativeness may inform, but not determine, future type-candidate selection. The validation principles are transferable in concept, whereas landmarks, thresholds, reference series, and biological conclusions must be rebuilt for each taxon. Most predictive variables in the present implementation are dimensionless ratios, so the workflow can operate primarily on scale-free relative morphology; calibrated images can add absolute-distance characters when available. Female-specific performance remains untested. Cross-observer landmark repeatability and transfer across acquisition or population domains also remain to be tested prospectively because the historical data do not retain the replicate measurements and blocking metadata needed for those analyses. The archive reproduces all statistical analyses from processed measurements; raw images and landmark coordinates are unavailable, while museum vouchers remain the means to independent remeasurement. Model output supports identification relative to available references, but species delimitation, type designation, and nomenclature remain expert- and integrative-taxonomic decisions.

## Data Availability

The complete computational archive contains the 70-specimen development matrix, projection matrix, exclusion record, 1400 repeated outer-fold predictions with fold and repeat identifiers, class scores, tuning results, selected variables and hyperparameters, leave-one-species-out decisions, specimen-cluster bootstrap results, benchmark outputs, executable R scripts, random seeds, package information, figures, session information, and SHA-256 checksums. The complete workflow was re-executed with R 4.3.3 end-to-end on Windows 11, completed with exit code 0, and generated 38 output files whose checksums were verified. The archive includes the regenerated outputs, the execution record, the session information, and the output-checksum manifest. It therefore reproduces the statistical stage from cleaned and harmonized character matrices. The original photographs and landmark-coordinate files are not retained in the present project archive and are no longer available from the corresponding author. Most underlying voucher and type specimens remain accessible in the institutional collections listed in Section 2 and may be independently re-examined, re-photographed, or remeasured with collection approval. A versioned Zenodo deposit of the final computational archive will be created before publication, and its DOI added to the published Data Availability Statement.

## Supplementary Materials

The following supporting information can be downloaded at: https://www.mdpi.com/article/10.3390/insects17090899/s1, Table S1: Definitions and analytical roles of morphometric characters used in the study; Table S2: Nested cross-validation performance metrics and specimen-cluster bootstrap confidence intervals; Table S3: Accuracy–coverage results across selective-classification thresholds; Table S4: Confusion matrix from repeated nested cross-validation; Table S5: Silhouette width and perturbation stability for alternative numbers of morphological groups; Table S6: Open-set projection results for taxonomically important specimens; Table S7: Summary of projection outcomes by specimen prefix or taxon code; Table S8: Records excluded before analysis because their source labels were explicitly uncertain; Table S9: Nested cross-validation performance of the pruned classification-tree identification key; Table S10: Confusion matrix for nested classification-tree predictions; Table S11: Out-of-fold calibration metrics for the multiclass and one-versus-rest classifiers; Table S12: Ten-bin reliability data for the multiclass classifier; Table S13: Leave-one-species-out open-set performance by omitted species, including resampling intervals; Table S14: Benchmark-model performance evaluated on identical outer-fold assignments; Table S15: Descriptive comparison of benchmark classifiers at approximately matched 82% coverage; Table S16: Specimen-cluster bootstrap confidence intervals for calibration, coverage, and conditional-accuracy metrics; Table S17: Specimen-cluster bootstrap confidence intervals for benchmark-model performance; Table S18: Paired specimen-cluster bootstrap differences between benchmark models; Table S19: Between-repeat sensitivity summaries for benchmark-model performance; Table S20: Equal-weight leave-one-species-out performance with biological specimen counts, specimen-repeat counts, effective prevalence, no-skill AUPRC baselines, and stratified specimen-cluster confidence intervals; Table S21: Calibration sensitivity using five equal-width and five equal-frequency bins, including prediction and unique-specimen counts; Table S22: Leave-one-species-out aggregation sensitivity comparing mean cutoff-normalized distance, median cutoff-normalized distance, and majority rejection across the five-fold-specific models, with empirical specimen-cluster bootstrap intervals.
